# Public Interest in Elective Orthopedic Surgery Following Recommendations During COVID-19: A Google Trends Analysis

**DOI:** 10.7759/cureus.12123

**Published:** 2020-12-17

**Authors:** Jonathan D Tijerina, Samuel A Cohen, Matthew J Parham, Christopher Debbaut, Landon Cohen, Milan Stevanovic, Rachel Lefebvre

**Affiliations:** 1 Bascom Palmer Eye Institute, University of Miami Hospital, Miami, USA; 2 Surgery, Stanford University School of Medicine, Palo Alto, USA; 3 Health and Research Policy, Stanford University School of Medicine, Palo Alto, USA; 4 Orthopaedic Surgery, Stanford University School of Medicine, Palo Alto, USA; 5 Orthopaedic Surgery, University of Southern California, Los Angeles, USA

**Keywords:** google trends, public interest, covid-19, coronavirus, orthopaedic surgery, arthroplasty, elective surgery

## Abstract

Introduction

Precautions issued by organizations such as the American Academy of Orthopaedic Surgeons (AAOS) recommending against any elective, or non-essential, surgical procedures have significantly affected healthcare resource utilization by the public during the severe acute respiratory syndrome coronavirus 2 (SARS-CoV-2) pandemic. In this study, we demonstrate the value of the Google Trends (GT) interface to characterize and monitor in real-time the response in public interest toward various elective orthopedic procedures.

Methods

Search volume databases were generated from January 2015 to May 2020 for keywords related to anterior cruciate ligament (ACL) reconstruction, elbow arthroplasty, hip arthroplasty, knee arthroplasty, and rotator cuff repair. To measure the immediate effects on public interest, the percent change was calculated from the AAOS, and Centers for Medicare and Medicaid Services (CMS) defined a 30-day pre-recommendations period to a 30-day post-recommendations period. To measure long-term effects, mean search volumes from January 1, 2015, to February 29, 2020, were compared to mean search volumes from March 1, 2020, to May 21, 2020.

Results

In the 30-day period following statements by the AAOS and CMS, interest in all search terms except “partial knee arthroplasty” decreased as follows: “ACL reconstruction” (-32.7%); “ACL repair” (-22.6%); “anterior cruciate ligament reconstruction” (-39.8%); “elbow arthroplasty” (-17.2%); “elbow joint replacement” (-15.1%); “total elbow arthroplasty” (-40.0%); “hip arthroplasty” (-23.0%); “hip replacement” (-41.2%); “total hip arthroplasty” (-23.0%); “knee arthroplasty” (-43.0%); “total knee arthroplasty” (-33.3%); “rotator cuff repair” (-34.2%); “rotator cuff surgery” (-50.6%); “shoulder arthroplasty” (-26.7%).

Discussion

GT data have previously demonstrated utility in characterizing and anticipating shifts in real-world healthcare utilization, making it an invaluable tool for physicians to anticipate and address the emerging needs of our patient population. Our study further illustrates the value of GT in localizing rapidly recovering interest in several of the most common elective orthopedic surgeries, enabling surgeons with up-to-date actionable data to guide the management of practices and healthcare facilities as the US slowly emerges from precautions endorsed during the COVID-19 pandemic.

## Introduction

The COVID-19 pandemic has drastically altered the medical landscape for both healthcare professionals and patients. One consequence of the unprecedented times has been a drastic shift in demand for elective orthopedic surgery [1]. Previously, the overall health of the economy and the stock market have both been identified as factors that correlate with public demand for elective surgical procedures [2-3]. However, there have never before been restrictions to elective procedures as severe and widespread as those that have been recommended during the COVID-19 pandemic. On March 13, 2020, the President of the United States declared COVID-19 to be a national emergency [4]. Shortly thereafter, the Centers for Medicare & Medicaid Services (CMS) and the American Academy of Orthopaedic Surgeons (AAOS) released recommendations for healthcare facilities and surgical practices to “ [delay] all elective surgeries, non-essential medical, surgical, and dental procedures” [5-6]

The recommendations issued by CMS and AAOS reverberated throughout the country, as the majority of healthcare systems ceased normal operations and shifted resources to the COVID-19 response. In addition to the official recommendations provided by CMS and AAOS, there has also been consistent high-profile press coverage regarding the implications of the COVID-19 pandemic on elective medical procedures, with the United States Surgeon General using Twitter, television appearances, and official press briefings to urge hospital systems to delay elective procedures [7]. Recent reports on a variety of surgical elective procedures indicate that this high-profile press coverage significantly impacts healthcare resource utilization by the public [8-10].

Google Trends (GT) is a free, open-source tool that has demonstrated utility in tracking both short-term and long-term variations in public interest regarding healthcare needs by offering near real-time updates for keywords that are entered into the Google search engine [11]. GT has previously been shown to be a valuable tool for gathering data and tracking interest in elective procedures for many different surgical specialties [12-16]. As states begin to gradually resume normal daily activities following the mandatory stay at home orders due to the pandemic, GT offers a free and intuitive way for surgeons to monitor public interest in orthopedic procedures [17].

In this study, we evaluate the utility of GT in monitoring variations in public interest regarding elective orthopedic procedures and subsequent real-world demand for orthopedic services during the COVID-19 pandemic. Specifically, we examine the ability of GT to characterize interest in anterior cruciate ligament (ACL) reconstruction, elbow arthroplasty, hip arthroplasty, knee arthroplasty, and rotator cuff repair in the United States both before and during the recommended cessation of elective and non-essential surgical interventions by the CMS and AAOS [18].

## Materials and methods

Public interest variations in popular elective orthopedic procedures following the release of official recommendations by the CMS and AAOS were collected. Databases of search volumes over time were collected for the following search terms: “ACL reconstruction”; “ACL repair”; “anterior cruciate ligament reconstruction”; “elbow arthroplasty”; “elbow joint replacement”; “total elbow arthroplasty”; “hip arthroplasty”; “hip replacement”; “total hip arthroplasty”; “knee arthroplasty”; “partial knee arthroplasty”; “total knee arthroplasty”; “rotator cuff repair”; “rotator cuff surgery”; and “shoulder arthroplasty”. Search terms were selected using the “related queries” feature of GT and researcher consensus [18-19]. We used GT’s customizable geographic and temporal filters to include results for searches within the United States from January 2015 to May 2020 for all selected terms.

After a search term is entered into the GT tool, GT generates outputs that describe the frequency of searches for a given search term relative to maximum popularity within a defined time period. The data are reported as relative search volume (RSV) values, which are computed as the ratio between searches for a given topic and the total amount of Google queries. RSV is reported on a scale of 0-100. An RSV of 100 indicates the peak popularity for a search term over the specified time period. An RSV of 0 indicates that at a certain time point, the proportion of queries for the term was less than 1% of its peak RSV (RSV 100).

Statistical analyses were carried out to illustrate both the short and long-term effects of these announcements. To measure short-term variations, the percent change from the 30-day period preceding the announcement from the CMS on March 18, 2020, was compared to the 30-day period after the announcement for each search term. To evaluate long-term variations of these recommendations, the mean search volume from January 2015 - February 2020 was compared to the mean search volume from March 2020 - May 2020 using two-sample t-tests. All statistical and trend analyses were performed using Microsoft Excel Version 15.21.1 (Redmond, WA) and SAS Studio Version 3.8 (Cary, NC).

## Results

Immediate effect on public interest

**Figure 1:**
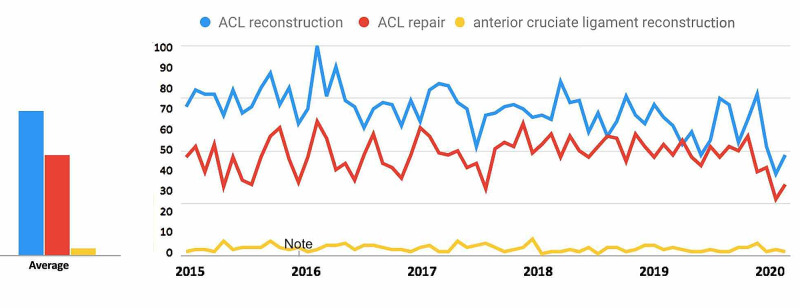
Temporal Trends in Public Interest in ACL Reconstruction-Related Search Terms Trends in the average monthly search volumes for ACL reconstruction-related search terms in the US from January 2015 to May 2020. Level of interest is expressed on a scale of 0% to 100%, with 100% interest reflecting the month with the highest search volume. (Blue = “ACL reconstruction”; Red = “ACL repair”; Yellow = “anterior cruciate ligament reconstruction”) ACL: anterior cruciate ligament

For search terms in the ACL Reconstruction-Related Search Terms category (Figure 1), all terms demonstrated a decrease in public interest following the official recommendations from the CMS and the AAOS in March 2020. For the search term “ACL reconstruction”, interest decreased from 53.7% in 30 days prior to March 19, 2020, to 36.1% during the 30 days post, a 32.7% decrease. For the search term “ACL repair”, interest decreased from 55.1% to 42.6%, a 22.6% decrease. For the search term “anterior cruciate ligament reconstruction”, interest decreased from 18.9% to 11.4%, a 39.8% decrease (Table 1).

**Table 1:** Percent Change in Public Interest in the 30-Day Period Following Official Recommendations by the CMS and AAOS Regarding Surgery During COVID-19 CMS: Centers for Medicare and Medicaid Services; AAOS: American Academy of Orthopaedic Surgeons

Category and Google Search Terms	Percent Change in Search Traffic in the Month Following CMS and AAOS Recommendations
ACL Reconstruction-Related Search Terms	
ACL reconstruction	-32.70%
ACL repair	-22.60%
Anterior cruciate ligament reconstruction	-39.80%
Elbow Arthroplasty-Related Search Terms	
Elbow arthroplasty	-17.20%
Elbow joint replacement	-15.10%
Total elbow arthroplasty	-40.00%
Hip Arthroplasty-Related Search Terms	
Hip arthroplasty	-23.00%
Hip replacement	-41.20%
Total hip arthroplasty	-22.80%
Knee Arthroplasty-Related Search Terms	
Knee arthroplasty	-43.00%
Partial knee arthroplasty	273.50%
Total knee arthroplasty	-33.30%
Rotator Cuff Repair-Related Search Terms	
Rotator cuff repair	-34.20%
Rotator cuff surgery	-50.60%
Shoulder arthroplasty	-26.70%

**Figure 2:**
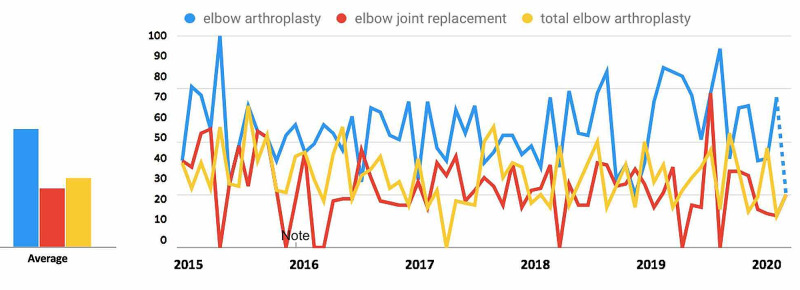
Temporal Trends in Public Interest in Elbow Arthroplasty-Related Search Terms Trends in the average monthly search volumes for elbow arthroplasty-related search terms in the US from January 2015 to May 2020. Level of interest is expressed on a scale of 0% to 100%, with 100% interest reflecting the month with the highest search volume. (Blue = “elbow arthroplasty”; Red = “elbow joint replacement”; Yellow = “total elbow arthroplasty”)

For search terms in the Elbow Arthroplasty-Related Search Terms category (Figure 2), all terms demonstrated a decrease in public interest following the official recommendations from the CMS and the AAOS in March 2020. For the search term “elbow arthroplasty”, interest decreased from 16.0% in the 30 days prior to March 19, 2020, to 13.2% during the 30days post, a 17.2% decrease. For the search term “elbow joint replacement”, interest decreased from 6.3% to 5.3%, a 15.1% decrease. For the search term “total elbow arthroplasty”, interest decreased from 18.2% to 11.0%, a 40.0% decrease (Table 1).

**Figure 3:**
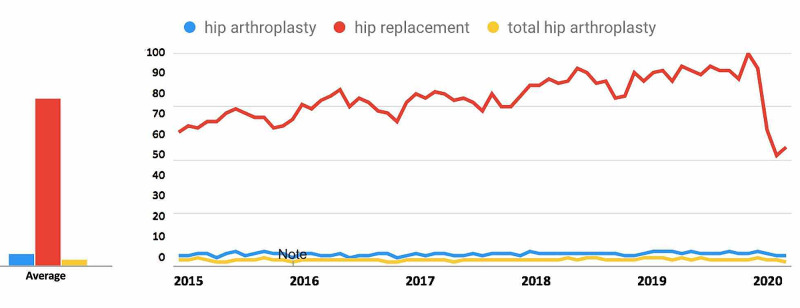
Temporal Trends in Public Interest in Hip Arthroplasty-Related Search Terms Trends in the average monthly search volumes for hip arthroplasty-related search terms in the US from January 2015 to May 2020. Level of interest is expressed on a scale of 0% to 100%, with 100% interest reflecting the month with the highest search volume. (Blue = “hip arthroplasty”; Red = “hip replacement”; Yellow = “total hip arthroplasty”)

For search terms in the Hip Arthroplasty-Related Search Terms category (Figure 3), all terms demonstrated a decrease in public interest following the official recommendations from the CMS and the AAOS in March 2020. For the search term “hip arthroplasty”, interest decreased from 48.7% in the 30 days prior to March 19, 2020, to 37.5% during the 30 days post, a 23.0% decrease. For the search term “hip replacement”, interest decreased from 68.9% to 40.5%, a 41.2% decrease. For the search term “total hip arthroplasty”, interest decreased from 37.8% to 29.2%, a 22.8% decrease (Table 1).

**Figure 4:**
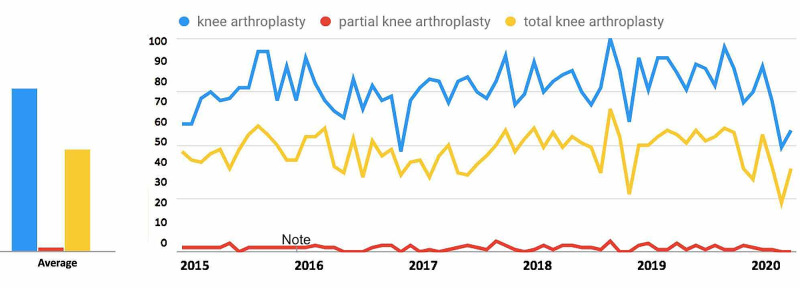
Temporal Trends in Public Interest Knee Arthroplasty-Related Search Terms Trends in the average monthly search volumes for knee arthroplasty-related search terms in the US from January 2015 to May 2020. Level of interest is expressed on a scale of 0% to 100%, with 100% interest reflecting the month with the highest search volume. (Blue = “knee arthroplasty”; Red = “partial knee arthroplasty”; Yellow = “total knee arthroplasty”)

For search terms in the Knee Arthroplasty-Related Search Terms category (Figure 4), “knee arthroplasty” and “total knee arthroplasty” demonstrated a decrease in public interest following the official recommendations from the CMS and AAOS in March 2020 while “partial knee arthroplasty” demonstrated an increase in public interest. For the search term “knee arthroplasty”, interest decreased from 55.0% in the 30 days prior to March 19, 2020, to 31.4% during the 30 days post, a 43.0% decrease. For the search term “total knee arthroplasty”, interest decreased from 53.5% to 35.7%, a 33.3% decrease. For the search term “partial knee arthroplasty”, interest increased from 3.3% to 12.5%, a 273.5% increase (Table 1).

**Figure 5:**
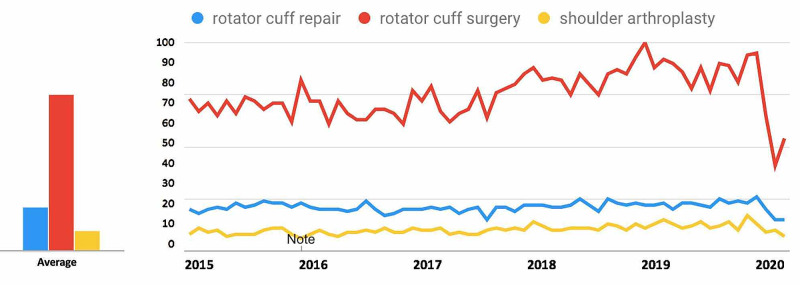
Temporal Trends in Public Interest in Rotator Cuff Repair-Related Search Terms Trends in the average monthly search volumes for rotator cuff repair-related search terms in the US from January 2015 to May 2020. Level of interest is expressed on a scale of 0% to 100%, with 100% interest reflecting the month with the highest search volume. (Blue = “rotator cuff repair”; Red = “rotator cuff surgery”; Yellow = “shoulder arthroplasty”)

For search terms in the Rotator Cuff Repair-Related Search Terms category (Figure 5), all terms demonstrated a decrease in public interest following the official recommendations from the CMS and AAOS in March 2020. For the search term “rotator cuff repair”, interest decreased from 50.2% in the 30 days prior to March 19, 2020, to 33.1% during the 30 days post, a 34.2% decrease. For the search term “rotator cuff surgery”, interest decreased from 69.1% to 34.1%, a 50.6% decrease. For the search term “shoulder arthroplasty”, interest decreased from 45.2% to 33.1%, a 26.7% decrease (Table 1).

Long-term effect on public interest

**Figure 6:**
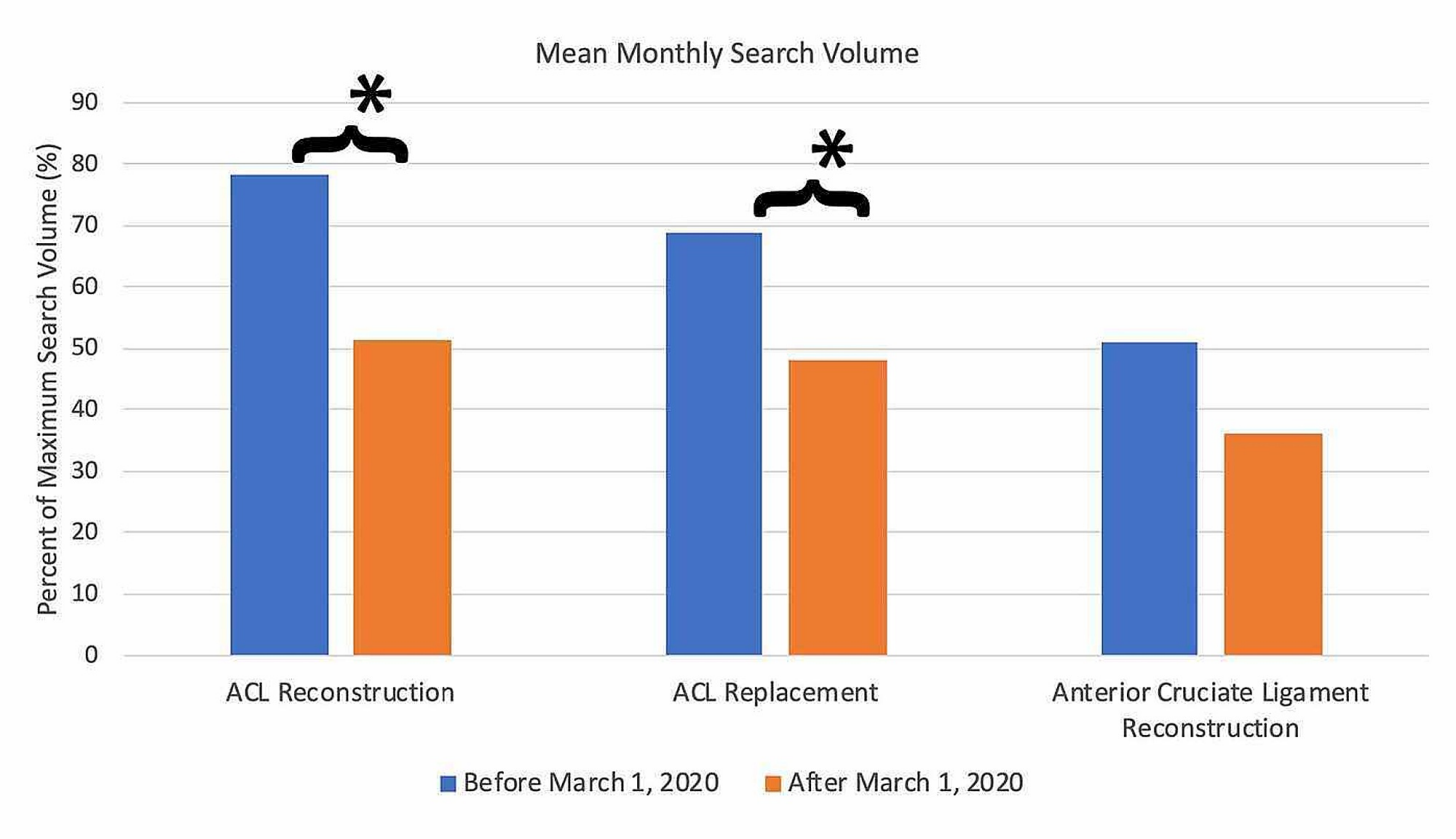
Long-Term Effects of Official Recommendations by the CMS and the AAOS on Public Interest in ACL Reconstruction-Related Search Terms Mean total search volumes before and after March 2020. Mean relative search volumes are compared between the period before announcements by the CMS and AAOS (January 2015 – Feb 2020) to the period afterward (March 2020 – May 2020). Pre- and post-announcement mean relative search volumes for each term were 78.37% and 51.33% for "ACL reconstruction" (p<0.0001*), 68.98% and 48.00% for "ACL replacement" (p = 0.012*), 51.05% and 36.33% for "anterior cruciate ligament reconstruction" (p = 0.136). Legend: * p < 0.05. CMS: Centers for Medicare and Medicaid Services; AAOS: American Academy of Orthopaedic Surgeons; ACL: anterior cruciate ligament

For search terms in the ACL reconstruction category (Figure 6), all terms demonstrated an overall decrease in public interest following the official recommendations from the CMS and the AAOS in March 2020. Pre- and post-announcement mean relative search volumes for each term in the ACL reconstruction-related category were 78.37% and 51.33% for "ACL reconstruction" (p<0.0001*), 68.98% and 48.00% for "ACL replacement" (p = 0.012*), and 51.05% and 36.33% for "anterior cruciate ligament reconstruction" (p = 0.136).

**Figure 7:**
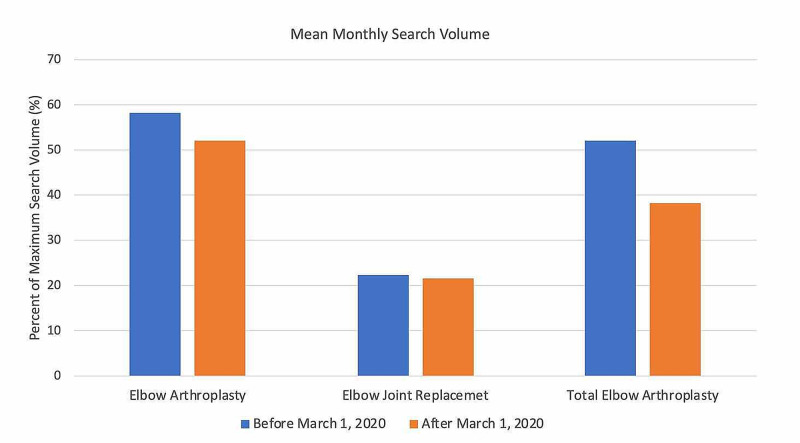
Long-Term Effects of Official Recommendations by the CMS and AAOS on Public Interest in Elbow Arthroplasty-Related Search Terms Mean total search volumes before and after March 2020. Mean relative search volumes are compared between the period before announcements by the CMS and AAOS (January 2015 – Feb 2020) to the period afterward (March 2020 – May 2020). Pre- and post-announcement mean relative search volumes for each term were 58.13% and 52.0% for "elbow arthroplasty" (p = 0.569), 22.35% and 21.67% for "elbow joint replacement" (p = 0.950), and 52.11% and 38.33% for "total elbow arthroplasty" (p = 0.221). Legend: * p < 0.05. CMS: Centers for Medicare and Medicaid Services; AAOS: American Academy of Orthopaedic Surgeons

For search terms in the elbow arthroplasty category (Figure 7), all terms demonstrated an overall decrease in public interest following the official recommendations from the CMS and AAOS in March 2020. Pre- and post-announcement mean relative search volumes for each term in the elbow arthroplasty-related category were 58.13% and 52.0% for "elbow arthroplasty" (p = 0.569), 22.35% and 21.67% for "elbow joint replacement" (p = 0.950), and 52.11% and 38.33% for "total elbow arthroplasty" (p = 0.221).

**Figure 8:**
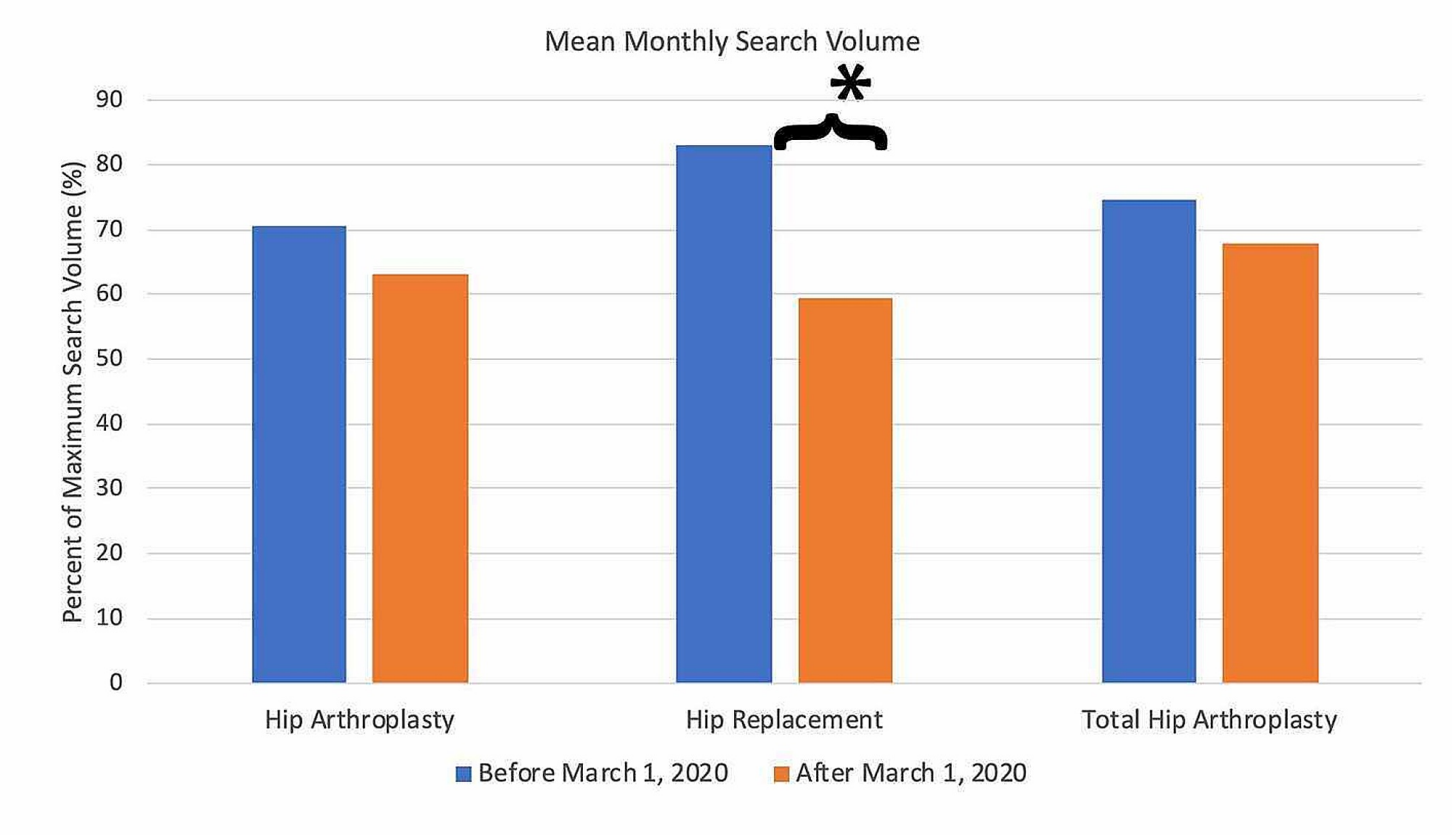
Long-Term Effects of Official Recommendations by the CMS and AAOS on Public Interest in Hip Arthroplasty-Related Search Terms Mean total search volumes before and after March 2020. Mean relative search volumes are compared between the period before announcements by the CMS and AAOS (January 2015 – Feb 2020) to the period afterward (March 2020 – May 2020). Pre- and post-announcement mean relative search volumes for each term were 70.63% and 63.33% for "hip arthroplasty" (p = 0.246), 83.00% and 59.33% for "hip replacement" (p < 0.0001*), and 74.61% and 68.00% for "total hip arthroplasty" (p = 0.405). Legend: * p < 0.05. CMS: Centers for Medicare and Medicaid Services; AAOS: American Academy of Orthopaedic Surgeons

For search terms in the hip arthroplasty category (Figure 8), all terms demonstrated an overall decrease in public interest following the official recommendations from the CMS and AAOS in March 2020. Pre- and post-announcement mean relative search volumes for each term in the hip arthroplasty-related category were 70.63% and 63.33% for "hip arthroplasty" (p = 0.246), 83.00% and 59.33% for "hip replacement" (p < 0.0001*), and 74.61% and 68.00% for "total hip arthroplasty" (p = 0.405).

**Figure 9:**
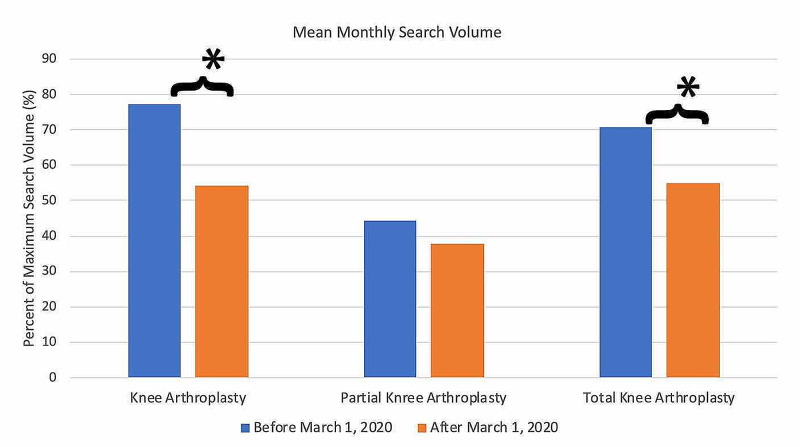
Long-Term Effects of Official Recommendations by the CMS and AAOS on Public Interest in Knee Arthroplasty-Related Search Terms Mean total search volumes before and after March 2020. Mean relative search volumes are compared between the period before announcements by the CMS and AAOS (January 2015 – Feb 2020) to the period afterward (March 2020 – May 2020). Pre- and post-announcement mean relative search volumes for each term were 77.32% and 54.33% for "knee arthroplasty" (p = 0.0003*), 44.40% and 37.67 for "partial knee arthroplasty" (p = 0.594), and 70.73% and 55.00% for "total knee arthroplasty" (p = 0.02*). Legend: * p < 0.05. CMS: Centers for Medicare and Medicaid Services; AAOS: American Academy of Orthopaedic Surgeons

For search terms in the knee arthroplasty category (Figure 9), all terms demonstrated an overall decrease in public interest following the official recommendations from the CMS and AAOS in March 2020. Pre- and post-announcement mean relative search volumes for each term in the knee arthroplasty-related category were 77.32% and 54.33% for "knee arthroplasty" (p = 0.0003*), 44.40% and 37.67 for "partial knee arthroplasty" (p = 0.594), and 70.73% and 55.00% for "total knee arthroplasty" (p = 0.020*).

**Figure 10:**
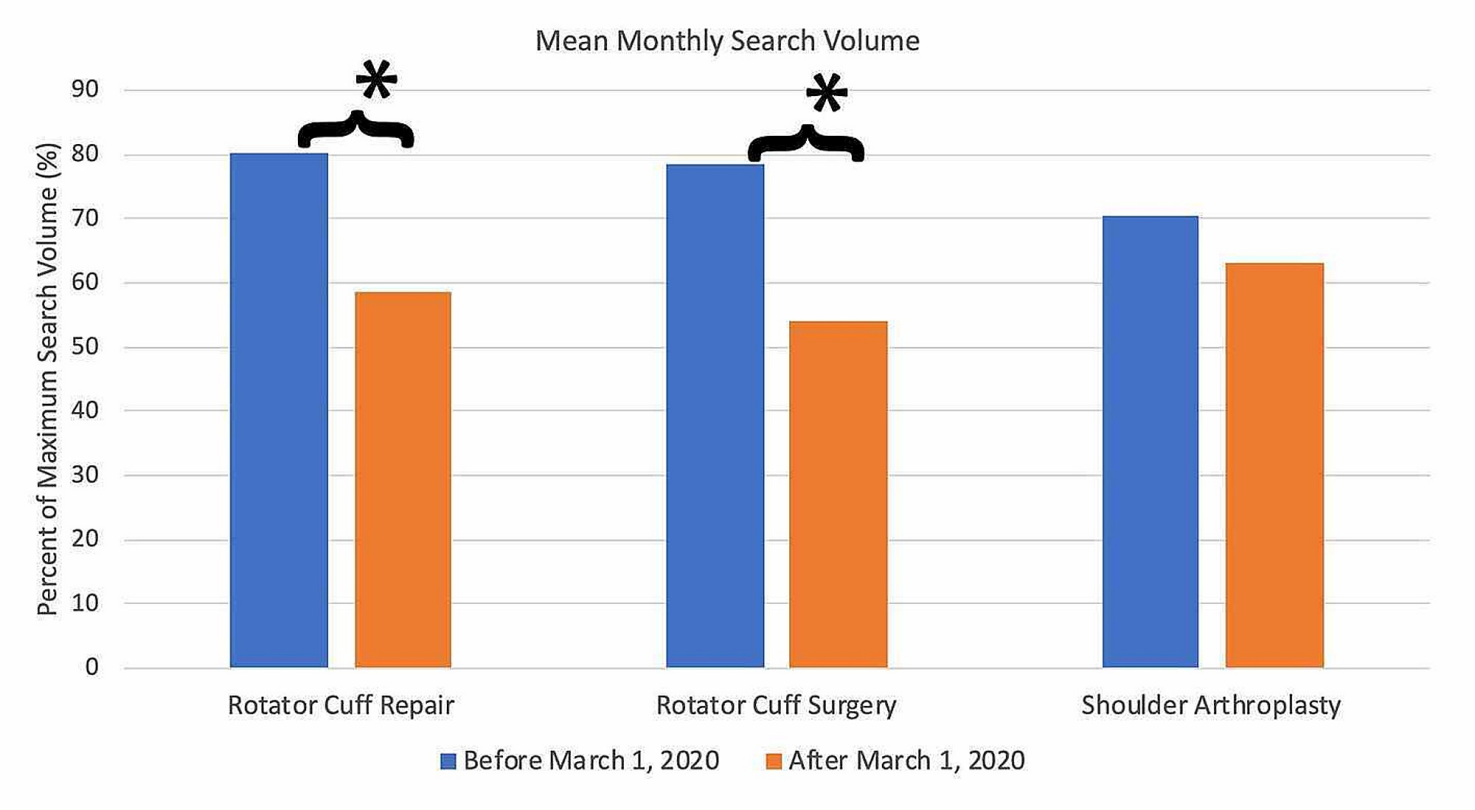
Long-term Effects of Official Recommendations by the CMS and AAOS on Public Interest in Rotator Cuff Surgery-Related Search Terms Mean total search volumes before and after March 2020. Mean relative search volumes are compared between the period before announcements by the CMS and AAOS (January 2015 – Feb 2020) to the period afterward (March 2020 – May 2020). Pre- and post-announcement mean relative search volumes for each term were 80.26% and 58.67% for "rotator cuff repair" (p < 0.0001*), 78.55% and 54.00% for "rotator cuff surgery" (p < 0.0001*), and 70.45% and 63.00% for "shoulder arthroplasty" (p = 0.387). Legend: * p < 0.05. CMS: Centers for Medicare and Medicaid Services; AAOS: American Academy of Orthopaedic Surgeons

For search terms in the rotator cuff repair category (Figure 10), all terms demonstrated an overall decrease in public interest following the official recommendations from the CMS and AAOS in March 2020. Pre- and post-announcement mean relative search volumes for each term in the rotator cuff surgery-related category were 80.26% and 58.67% for "rotator cuff repair" (p < 0.0001*), 78.55% and 54.00% for "rotator cuff surgery" (p < 0.0001*), and 70.45% and 63.00% for "shoulder arthroplasty" (p = 0.387).

Overall popularity of search terms

Among the search terms analyzed in the ACL Reconstruction-Related Search Terms category, “ACL reconstruction” had the highest overall interest, followed by “ACL repair” and “anterior cruciate ligament reconstruction” (Figure 1). When examining search terms in the Elbow Arthroplasty-Related Search Terms category, “elbow arthroplasty” had the highest overall interest, followed by “total elbow arthroplasty” and “elbow joint replacement” (Figure 2). Among the search terms analyzed in the Hip Arthroplasty-Related Search Terms category, “hip replacement” had the highest overall interest, followed by “hip arthroplasty” and “total hip arthroplasty” (Figure 3). When examining search terms in the Knee Arthroplasty-Related Search Terms category, “knee arthroplasty” had the highest overall interest, followed by “total knee arthroplasty” and “partial knee arthroplasty” (Figure 4). Among the search terms analyzed in the Rotator Cuff Repair-Related Search Terms category, “rotator cuff surgery” had the highest overall interest, followed by “rotator cuff repair” and “shoulder arthroplasty” (Figure 5).

Interest in the USA by state

Interest was reported by GT on a scale of 0% to 100%, where 100% interest reflects the highest level of interest for a given search term in a country or region, with the percentages for other geographic regions reflecting interest relative to the peak level. The five subregions that demonstrated the highest level of interest for each search term are reported unless there were insufficient data to report five or more subregions. If there is insufficient data to report five subregions, levels of interest for all available subregions are reported. Geographic variation for the most popular search term in each category was analyzed.

**Figure 11:**
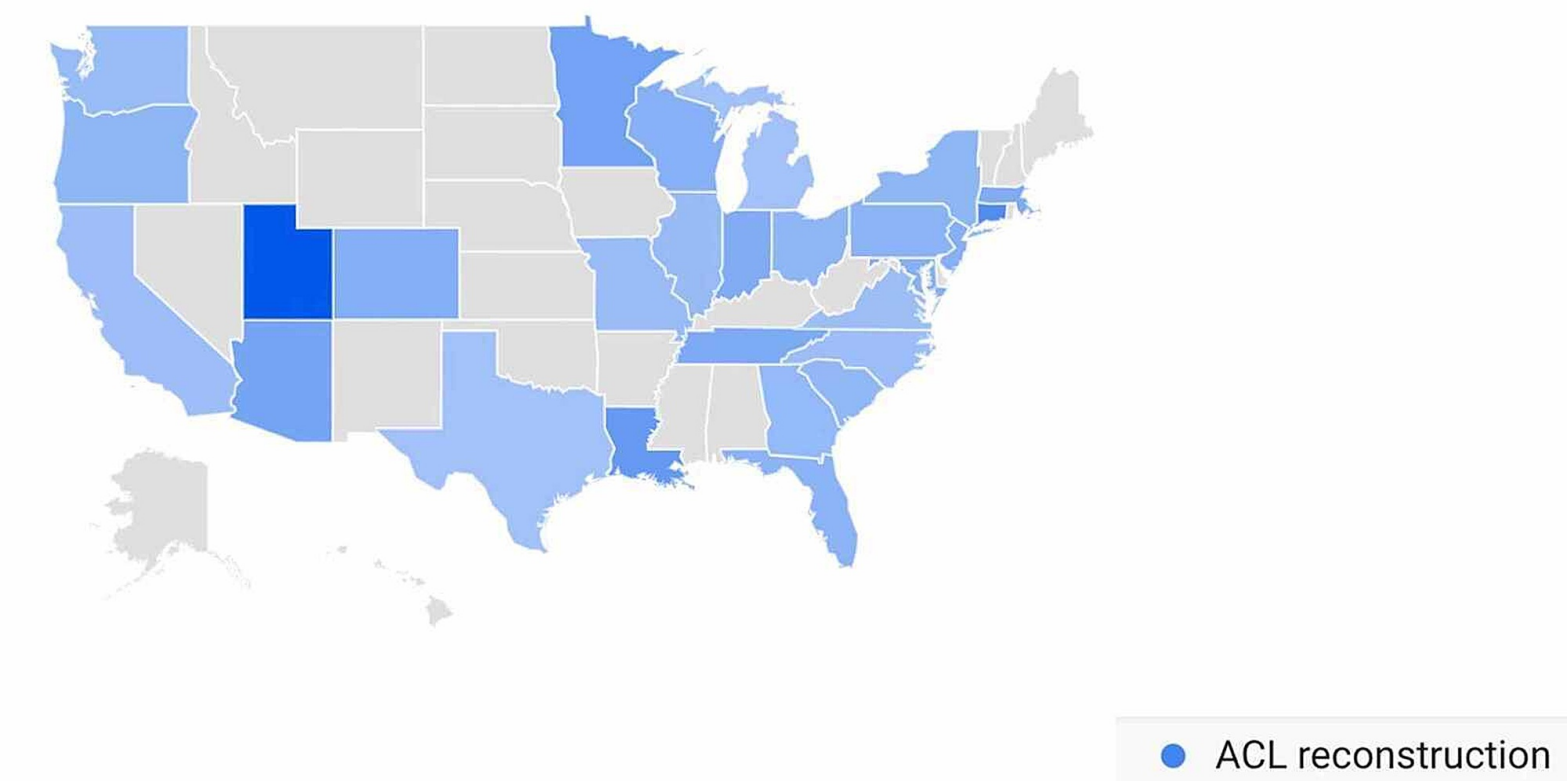
Geographic Trends in Public Interest in ACL Reconstruction (Pre-CMS and AAOS Recommendations) United States map illustrating the states with the highest Google Trends search volumes for ACL reconstruction. In the 30 days prior to March 19, 2020; interest in “ACL reconstruction” was highest in Utah (100%), Connecticut (54%), Louisiana (43%), Minnesota (36%), and Arizona (34%). Note: A darker shade indicates greater search volumes. CMS: Centers for Medicare and Medicaid Services; AAOS: American Academy of Orthopaedic Surgeons; ACL: anterior cruciate ligament

**Figure 12:**
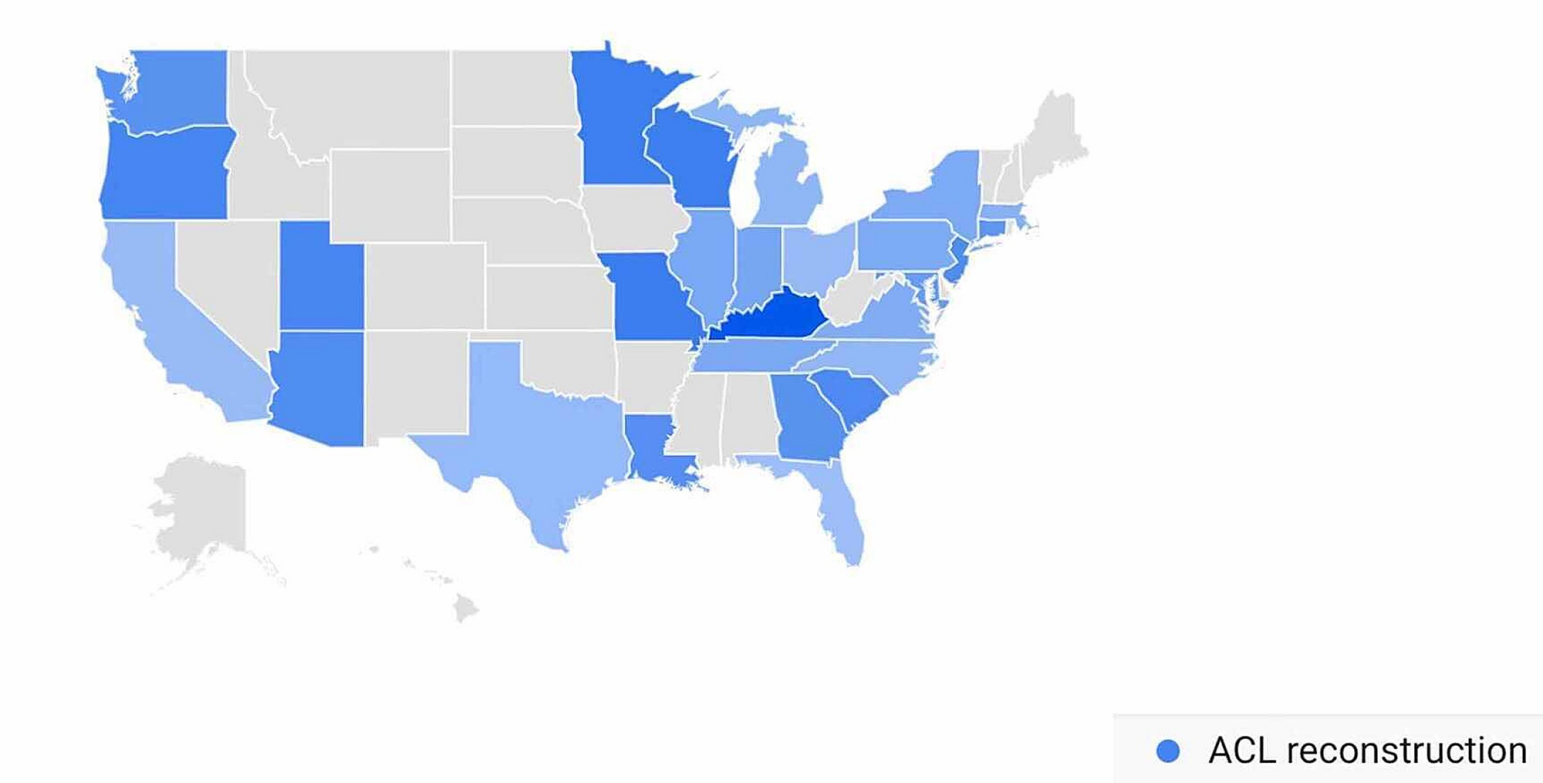
Geographic Trends in Public Interest in ACL Reconstruction (Post-CMS and AAOS Recommendations) United States map illustrating the states with the highest Google Trends search volumes for ACL reconstruction. In the 30 days post-announcement, interest in “ACL reconstruction” was highest in Kentucky (100%), Missouri (68%), Wisconsin (67%), Minnesota (67%), and Oregon (61%). Note: A darker shade indicates greater search volumes. CMS: Centers for Medicare and Medicaid Services; AAOS: American Academy of Orthopaedic Surgeons; ACL: anterior cruciate ligament

In the ACL Reconstruction-Related Search Terms category, geographic variation in public interest for “ACL reconstruction” was observed when comparing pre- and post-CMS/AAOS announcement search volumes. In the 30 days prior to March 19, 2020, interest in the search term “ACL reconstruction” was highest in Utah (100%), followed by Connecticut (54%), Louisiana (43%), Minnesota (36%), and Arizona (34%) (Figure 11). In the 30-days post-announcement, interest was highest in Kentucky (100%), Missouri (68%), Wisconsin (67%), Minnesota (67%), and Oregon (61%) (Figure 12).

**Figure 13:**
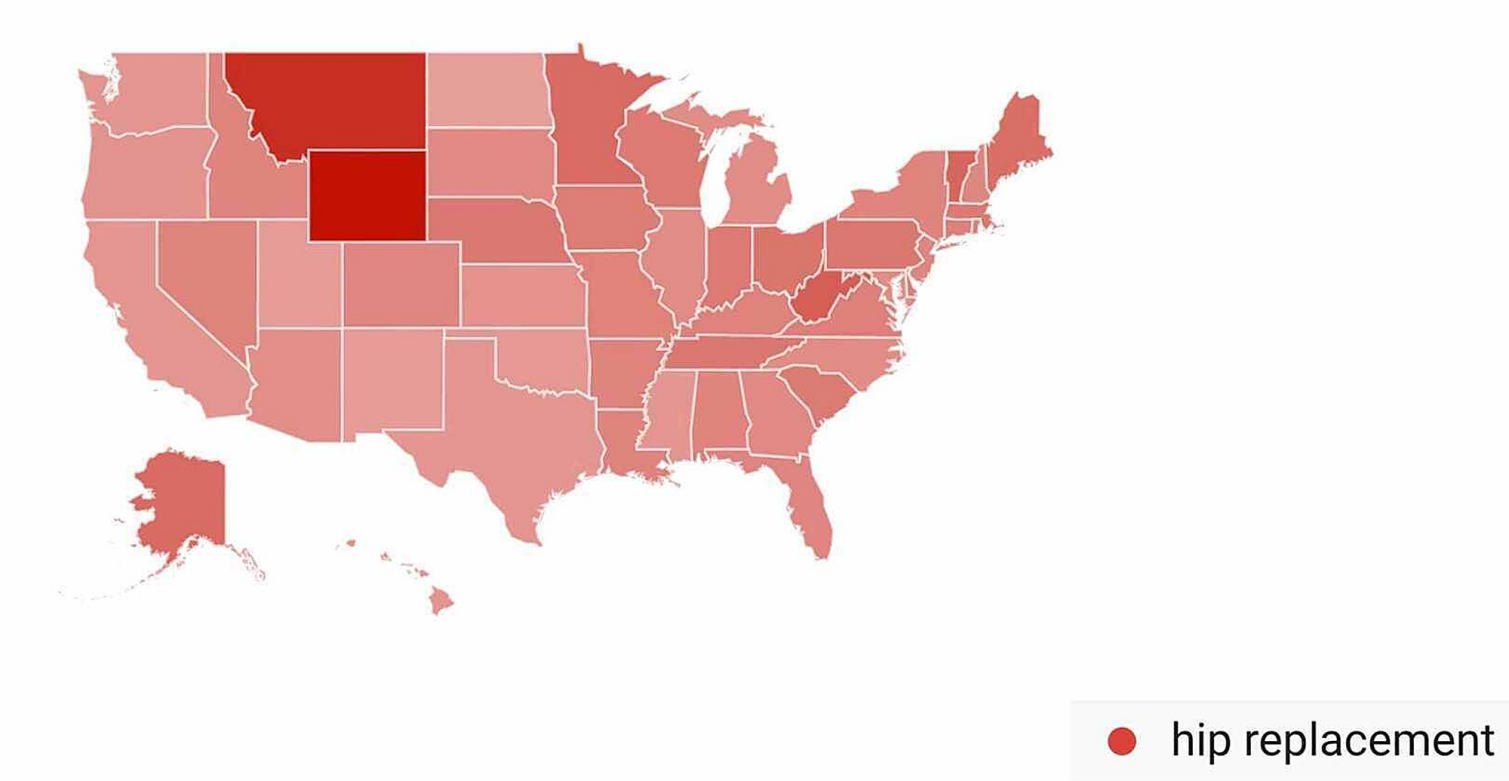
Geographic Trends in Public Interest in Hip Replacement (Pre-CMS and AAOS Recommendations) United States map illustrating the states with the highest Google Trends search volumes for hip replacement. In the 30 days prior to March 19, 2020, interest in “hip replacement” was highest in Wyoming (100%), Montana (84%), West Virginia (56%), Vermont (53%), and Minnesota (50%). Note: A darker shade indicates greater search volumes. CMS: Centers for Medicare and Medicaid Services; AAOS: American Academy of Orthopaedic Surgeons

**Figure 14:**
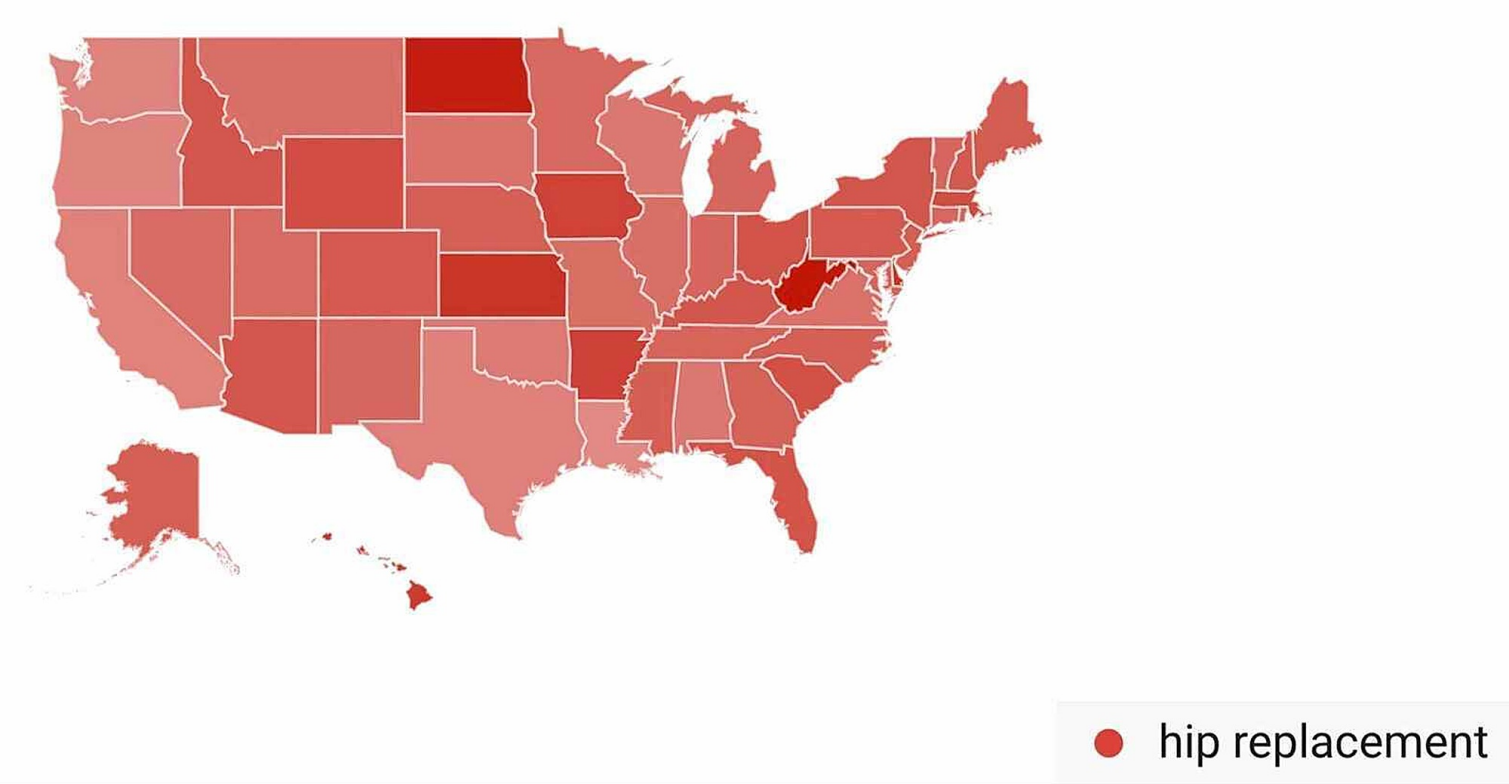
Geographic Trends in Public Interest in Hip Replacement (Post-CMS and AAOS Recommendations) United States map illustrating the states with the highest Google Trends search volumes for hip replacement. In the 30 days post-announcement; interest in “hip replacement” was highest in Washington, D.C. (100%), West Virginia (97%), North Dakota (93%), Kansas (81%), and Delaware (79%). Note: A darker shade indicates greater search volumes. CMS: Centers for Medicare and Medicaid Services; AAOS: American Academy of Orthopaedic Surgeons

In the Hip Arthroplasty-Related Search Terms category, geographic variation in public interest for “hip replacement” was observed when comparing pre- and post-CMS/AAOS announcement search volumes. In the 30 days prior to March 19, 2020, interest in the search term “hip replacement” was highest in Wyoming (100%), followed by Montana (84%), West Virginia (56%), Vermont (53%), and Minnesota (50%) (Figure 13). In the 30-days post-announcement, interest was highest in Washington, D.C. (100%), West Virginia (97%), North Dakota (93%), Kansas (81%), and Delaware (79%) (Figure 14).

**Figure 15:**
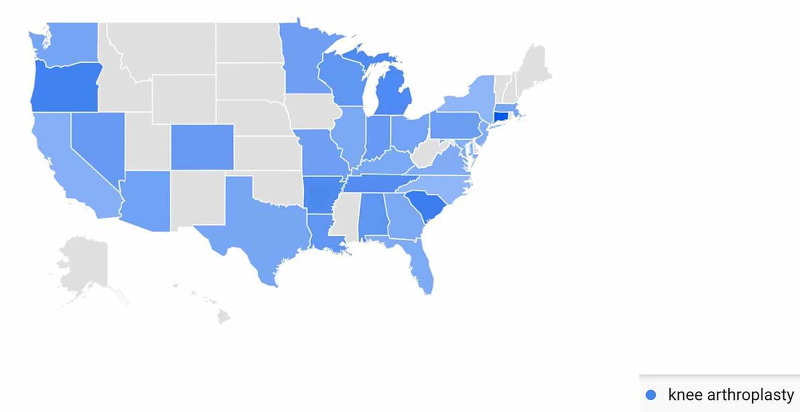
Geographic Trends in Public Interest in Knee Arthroplasty (Pre-CMS and AAOS Recommendations) United States map illustrating the states with the highest Google Trends search volumes for knee arthroplasty. In the 30 days prior to March 19, 2020, interest in “knee arthroplasty” was highest in Connecticut (100%), South Carolina (68%), Oregon (64%), Michigan (57%), and Arkansas (56%). Note: A darker shade indicates greater search volumes. CMS: Centers for Medicare and Medicaid Services; AAOS: American Academy of Orthopaedic Surgeons

**Figure 16:**
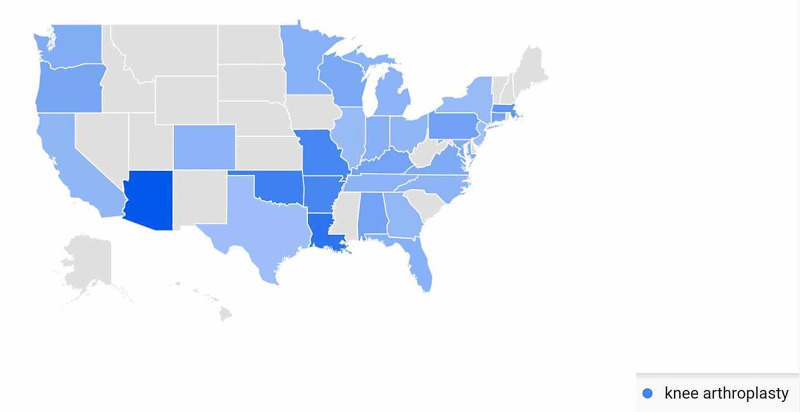
Geographic Trends in Public Interest in Knee Arthroplasty (Post-CMS and AAOS Recommendations) United States map illustrating the states with the highest Google Trends search volumes for knee arthroplasty. In the 30 days post-announcement; interest in “knee arthroplasty” was highest in Arizona (100%), Louisiana (76%), Oklahoma (66%), Missouri (61%), and Arkansas (60%). Note: A darker shade indicates greater search volumes. CMS: Centers for Medicare and Medicaid Services; AAOS: American Academy of Orthopaedic Surgeons

In the Knee Arthroplasty-Related Search Terms category, geographic variation in public interest for “knee arthroplasty” was observed when comparing pre- and post-CMS/AAOS announcement search volumes. In the 30-days prior to March 19, 2020, interest in the search term “knee arthroplasty” was highest in Connecticut (100%), followed by South Carolina (68%), Oregon (64%), Michigan (57%), and Arkansas (56%) (Figure 15). In the 30-days post-announcement, interest was highest in Arizona (100%), Louisiana (76%), Oklahoma (66%), Missouri (61%), and Arkansas (60%) (Figure 16).

**Figure 17:**
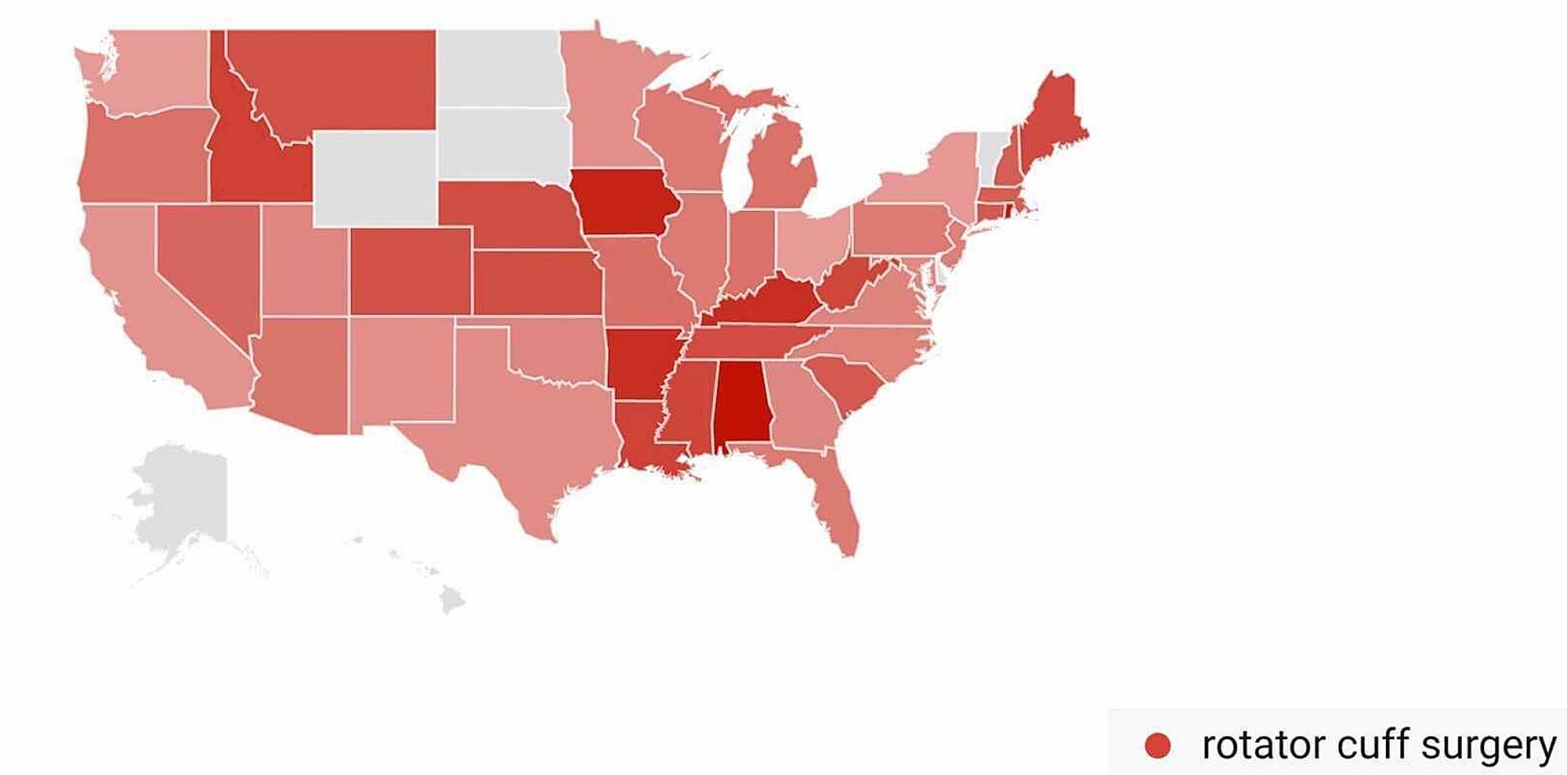
Geographic Trends in Public Interest in Rotator Cuff Surgery (Pre-CMS and AAOS Recommendations) United States map illustrating the states with the highest Google Trends search volumes for rotator cuff surgery. In the 30 days prior to March 19, 2020; interest in “rotator cuff surgery” was highest in Alabama (100%), Rhode Island (93%), Iowa (90%), Kentucky (84%), and Arkansas (84%). Note: A darker shade indicates greater search volumes. CMS: Centers for Medicare and Medicaid Services; AAOS: American Academy of Orthopaedic Surgeons

**Figure 18:**
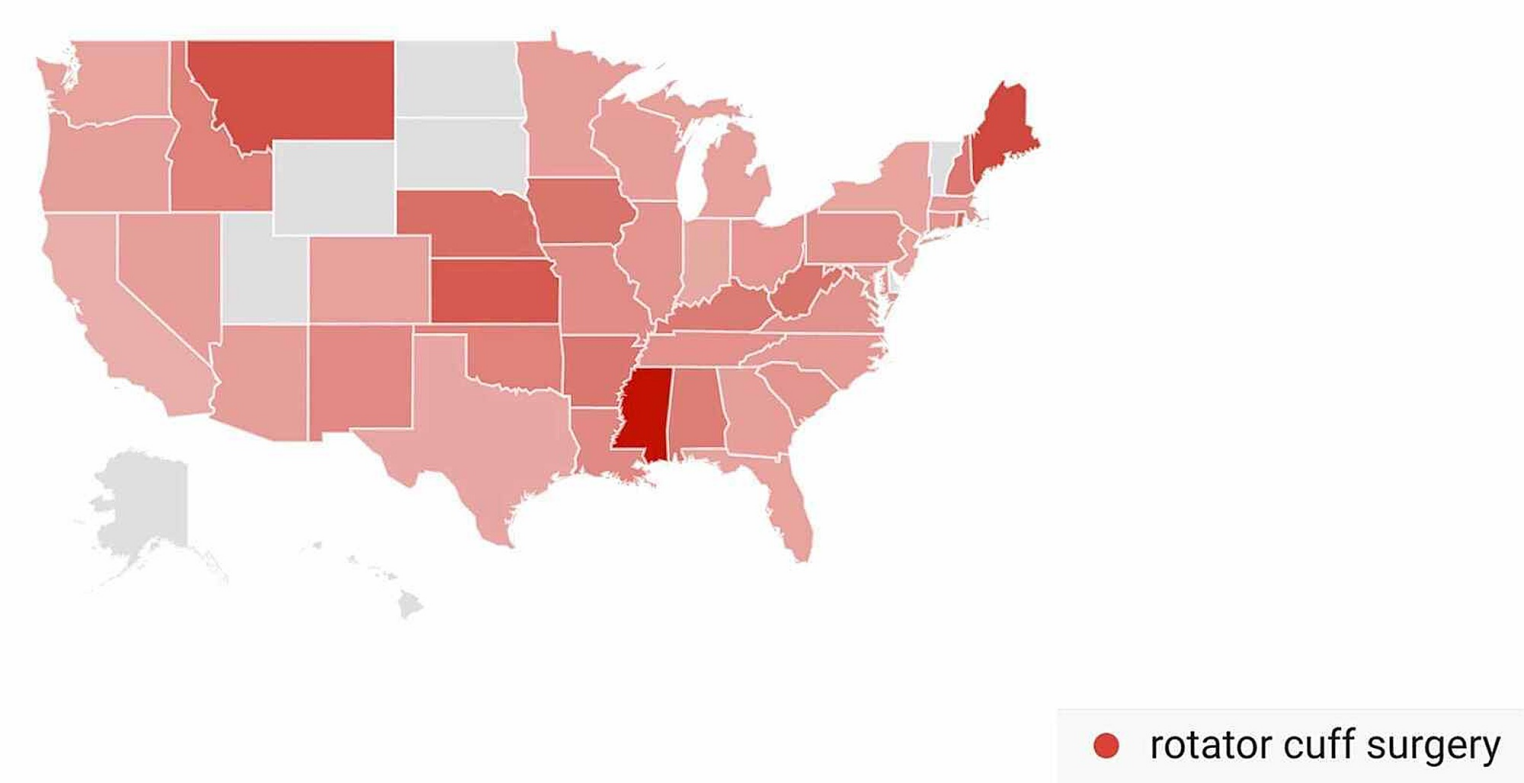
Geographic Trends in Public Interest in Rotator Cuff Surgery (Post-CMS and AAOS Recommendations) United States map illustrating the states with the highest Google Trends search volumes for rotator cuff surgery. In the 30 days post-announcement; interest in “rotator cuff surgery” was highest in Mississippi (100%), Maine (68%), Montana (65%), Washington, D.C. (61%), and Kansas (59%). Note: A darker shade indicates greater search volumes. CMS: Centers for Medicare and Medicaid Services; AAOS: American Academy of Orthopaedic Surgeons

In the Rotator Cuff Repair-Related Search Terms category, geographic variation in public interest for “rotator cuff surgery” was observed when comparing pre- and post-CMS/AAOS announcement search volumes. In the 30 days prior to March 19, 2020, interest in the search term “rotator cuff surgery” was highest in Alabama (100%), followed by Rhode Island (93%), Iowa (90%), Kentucky (84%), and Arkansas (84%) (Figure 17). In the 30-days post-announcement, interest was highest in Mississippi (100%), Maine (68%), Montana (65%), Washington, D.C. (61%), and Kansas (59%) (Figure 18).

There was insufficient data to report state-by-state variations in public interest in the search term “elbow arthroplasty” in the 30 days pre and post the CMS/AAOS announcement.

## Discussion

The steep decline in elective surgical procedures in the United States in response to recommendations by the CMS and AAOS to postpone all elective and/or non-emergent surgeries in March 2020 is unprecedented [5-6]. Social distancing requirements have forced many healthcare professionals, including orthopedic surgeons, to seek out alternative methods of communicating with and educating patients. Telehealth appointments have exploded in popularity since the pandemic began and may become more commonplace in the future, with patients previously reporting increased satisfaction, increased convenience, and decreased costs associated with online rather than in-person visits [20-21]. Our study suggests that in addition to monitoring telehealth orthopedic inquiries, GT may be an effective tool for real-time monitoring of patient interest in orthopedic procedures during the COVID-19 pandemic as states continue to progress through their phased approaches to resuming normal daily activities [17].

Decreased search volumes for each of the included search terms in the 30-day-period after official press releases from the CMS and AAOS could have long-lasting implications. When comparing the pre-event 30-day period to the 30-day period following the press releases, 14 out of 15 of the relevant search terms had lower search volumes (Table 1). This demonstrated decrease in public interest may point to an enduring rather than a temporary phenomenon, as several previous studies in surgical literature suggest that early trends detected by GT can anticipate acutely developing patterns before they prove to be long-term [9-10]. Signs of pronounced, persistent decreases in GT search volumes may already be emerging for the search terms ACL reconstruction, ACL replacement, hip replacement, knee arthroplasty, total knee arthroplasty, rotator cuff repair, and rotator cuff surgery, where there were significant decreases in public interest when comparing mean search volumes from January 2015 - February 2020 to search volumes from March 2020 - May 2020 (Figures 6-10).

Previous studies that demonstrate a correlation between GT and actual healthcare utilization suggest that GT may prove beneficial in tracking both significant decreases in public interest in orthopedic procedures during the COVID-19 pandemic but also significant increases in these procedures as states resume normal daily activities. For example, several search terms, including “ACL reconstruction”, “ACL repair”, “total elbow arthroplasty”, “hip replacement”, “knee arthroplasty”, “total knee arthroplasty”, and “rotator cuff surgery” demonstrate increased Internet search traffic during the months of April and May, correlating with anticipated and actualized phase 1 re-openings around the United States. It remains to be seen, though, if the increase in search traffic will result in a return to the pre-pandemic levels observed from January 2015 - February 2020. The correlation between increased Google search traffic and United States procedure volumes for several different types of surgical procedures has been well-documented by Tijerina et al. [12-16]. The fact that GT has been shown to track actual volumes of medical procedures, rather than just theoretical interest in procedures, means that GT is a tool that could provide immense value to orthopedic surgeons and healthcare facilities as they anticipate and respond to varying public interest in elective orthopedic procedures as states cycle between various COVID-related restrictions. The utility of this information is becoming even more relevant as the AAOS and CMS release guidelines and considerations for elective orthopedic surgeries [22-24].

A state-by-state analysis of GT search volumes can also provide insight as to when to expect public interest in various orthopedic procedures to return to pre-pandemic levels. States are advancing through phased returns to normal daily activities at very different rates, and examining trends in public interest in orthopedic procedures in states that reopen sooner may inform public interest in states that are slower to reopen. While all procedures we studied showed geographic variation when comparing search volumes pre- and post-CMS and AAOS official recommendations, knee arthroplasty was a procedure with a very specific geographic shift in GT search volumes. In the 30 days post-CMS and AAOS official recommendations, state-by-state comparisons showed a national shift in search traffic for “knee arthroplasty” away from states with more restrictive stay-at-home orders such as Connecticut and Michigan and toward states with less restrictive stay-at-home orders such as Oklahoma and Arizona (Figure 16) [17]. A similar trend was seen with public interest in “hip replacement”, where the largest relative increase in search traffic post-CMS and AAOS recommendations was seen in North Dakota, a state where no stay-at-home order was issued (Figure 14) [17]. The largest relative increase in public interest in “rotator cuff surgery” post-CMS and AAOS recommendations was seen in Mississippi, a state whose shelter in place order expired earlier than most other states (Figure 18) [17].

These findings point to a possible relationship between public perception of the coronavirus (as indicated by a state’s messaging surrounding the virus) and a willingness to proceed with elective orthopedic surgery. People who live in a state with harsher stay-at-home orders, for example, may be more cautious about leaving home to continue with elective surgery if they are worried that elective surgery may substantially increase their risk of acquiring COVID-19; however, citizens who live in a state whose governor minimizes the risk of the virus may feel more inclined to continue with elective surgery. This information may be useful when attempting to project interest in orthopedic procedures if a “second wave” or “third wave” of COVID-19 occurs in the coming months and states need to retreat to previous phases of their return to normal daily activities. In fact, many of the states that avoided large outbreaks of COVID-19 in the beginning of the pandemic later saw an uptick in infections. The governor of Texas, a state that wasn’t initially considered a “hot spot” for COVID-19 infections, issued a statement on June 25, 2020, indicating that he was suspending elective surgeries in the state’s biggest cities due to a recent surge in cases [25]. As public messaging surrounding the virus changes rapidly at both state and federal levels, subsequent trends in interest in elective orthopedic procedures should be further examined.

There are several limitations to our study. First, GT provides very limited information about the demographic information of the users whose data has been collected in this research. GT also uses relative search volume (RSV) rather than absolute search volume data, which prevents further analysis regarding the unique number of users and/or searches reflected in the GT output. Additionally, while Google does capture the majority of Internet search traffic worldwide, there are other search engines that patients may use to inquire about medical information [26]. Finally, while the majority of search terms did have decreased search volumes both in the short-term and long-term after public announcements by the CMS and AAOS, some terms did not. Further studies should attempt to decipher the reason why there was increased interest in some procedures, such as partial knee arthroplasty, but decreased interest in other procedures during the COVID-19 pandemic. We believe that despite these limitations, GT data can provide unique insight that will enable orthopedic surgeons and healthcare systems to better monitor and respond to patients’ evolving interests in elective orthopedic procedures as states across the country grapple with how to best resume normal daily activities.

## Conclusions

In conclusion, this study illustrates the utility and convenience of the GT tool in describing rapidly evolving trends in public interest regarding several common elective orthopedic procedures in the United States during the COVID-19 pandemic. These trends can provide valuable information to orthopedic surgeons and healthcare systems attempting to gauge public interest in demand for surgical procedures as states begin to reopen across the country and beyond. GT provides real-time insight into trends in public interest at the state, county, and city levels that can allow orthopedic practices to better allocate resources and anticipate patient needs as the desire and capacity for many elective procedures gradually increase across the country.
